# Incorporation of alpha-fetoprotein(AFP) into subclassification of BCLC C stage hepatocellular carcinoma according to a 5-year survival analysis based on the SEER database

**DOI:** 10.18632/oncotarget.13232

**Published:** 2016-11-09

**Authors:** Nan Zhang, Jiajia Gu, Li Yin, Jing Wu, Ming-yu Du, Kai Ding, Teng Huang, Xia He

**Affiliations:** ^1^ The Fourth Clinical School of Nanjing Medical University, Nanjing, China; ^2^ Department of Radiation Oncology, Jiangsu Cancer Hospital and Jiangsu Institute of Cancer Research, Nanjing, China; ^3^ Jiangsu Key Laboratory of Molecular and Translational Cancer Research, Nanjing, China; ^4^ Xuzhou Medical University, Xuzhou, China

**Keywords:** AFP, hepatocellular carcinoma, BCLC, SEER

## Abstract

**Purpose:**

To evaluate the effect of serum alpha-fetoprotein(AFP) on prognosis of patients with hepatocellular carcinoma (HCC) and put forward a proposal to modify BCLC staging system and the recommended treatment of patients with stage C.

**Results:**

AFP positive was an independent poor prognostic factor of HCC. Race, pathological grade, T stage, M stage were also regarded to be significant predicted factors for poorer prognosis. When combining AFP status with AJCC stage, patients with A1 disease had a worse prognosis compared with those with A0 disease within each stage. Patients with A1 disease of each T/N stage had a worse prognosis than patients with A0 disease of the respective stage, and the prognosis of patients with A1 disease with lower T stages was worse or similar to that of patients with A0 disease of higher T stages.

**Materials and Methods:**

We performed a retrospective study of all patients histologically diagnosed HCC from January 1, 2004, through December 31, 2008, from the SEER database.

**Conclusions:**

AFP can be used as a subclassification index to modify the AJCC staging system of HCC. Since BCLC stage is the most widely used staging system, we recommend routine pre-treatment AFP testing as standard of care in HCC and incorporate AFP status into the BCLC staging system to modify the recommended treatment of patients with stage C.

## INTRODUCTION

Liver cancer is a threat to public health and a major cause of cancer-related deaths in our country and many other parts of the world, 90% of which is hepatocellular carcinoma (HCC) [1]. About 6.12 new cases and 4.52 deaths occur annually per 100,000 people in United States of America according to GLOBOCAN2012 (http://globocan.iarc.fr/Pages/Map.aspx#).

Successful management of cancer depends on adequate therapeutic protocols, evaluation of curative effect and prognostication based on accurate staging in clinical practice. The clinicians has identified the potential limitation of pure anatomical staging. Optimal treatment depends on the anatomical staging (i.e. traditional tumor-node-metastasis (TNM) stage, the underlying liver function, and the general state of the patient [2]. To date, the classification criteria of HCC is still controversial. The TNM system, the most widely adopted standard staging system of all cancers, was considered not informative for HCC with regard to treatment guidance efficacy and prognosis accuracy [3]. Novel combined-staging systems using tumor and residual liver function factors have been proposed, such as the Cancer of the Italian Liver Program (CLIP) [4], the Barcelona Clinic Liver Cancer (BCLC) [5], the Japanese Intergrated Staging (JIS) [6] and the Hong Kong Liver Cancer [7]. Among them, the BCLC is a reference classification system of HCC in the West, which is based on randomized studies and recommend treatment modality. Nonetheless, the BCLC is still criticized for its lack of universal applicability. Kwang et al. demonstrated that the 5-year survival rate of the the population that received therapy according to the BCLC treatment algorithm was better only for the early stages (0, A) [8].The reason is not obvious. Taken together, a reconsideration of treatment strategies is urgent for the management of HCC.

Despite the disadvantage of low sensitivity, low specificity and limited accuracy in HCC early diagnosis, serum alpha-fetoprotein (AFP) has still been recommended as a biomarker to evaluate prognosis and monitor recurrence following treatment for HCC in clinical practice [9–12]. Accordingly, to address the above limitations, we intend to use AFP as a subclassification criterion to modify the BCLC stage. We used the National Cancer Institutes Surveillance, Epidemiology, and End Results (SEER) database to analyze the survival outcomes after incorporating AFP into the AJCC staging.

## RESULTS

### Clinicopathologic characteristics of patients with A0 and A1 stage

During the 5-year study period, 12908 records were enrolled from the SEER database, including 11999 hepatocellular carcinoma (HCC), 796 intrahepatic cholangiocarcinoma (ICC) and 113 combined HCC and ICC. Analyzing AFP level on overall survival (OS) and disease-special survival (DSS) based on different histological types by univariate analysis showed that A0 stage patients had an increased 5-year OS and DSS in HCC group (Table 1). However, similar difference hasn't been seen in other subgroups (ICC and combined). Therefore, we choose all patients with HCC to continue our study. Patient demographics and clinicopathologic features are summarized in Table 2.

**Table 1:** Univariate survival analysis of AFP on OS and DSS based on different histological types via standard Kaplan–Meier estimates

Variable	*n*	5-year OS (%)	*P* value	5-year DSS (%)	*P* value
**HCC**					
A0	2660	31	< 0.001	38	< 0.001
A1	9339	15		19	
**ICC**					
A0	544	11	.230	12	.236
A1	252	7		9	
**Combined**					
A0	35	15	.742	24	.751
A1	78	18		23	

Abbreviations: OS, overall survival. DSS, disease-specific survival. HCC, hepatocellular carcinoma. ICC, intrahepatic cholangiocarcinoma. Combined, HCC and ICC.

**Table 2:** Comparison of patients with A0 and A1 disease

	NO.(%) of patients			*P* value (χ^2^ or Student's *t*-test)
Characteristic	All (*N* = 11999)	A0 (*N* = 2660)	A1 (*N* = 9339)	
**Age, y**				
≤ 45	733 (6.1)	186 (7.0)	547 (5.9)	.031
> 45	11266 (93.9)	2474 (93.0)	8792 (94.1)	
**Sex**				
Male	9344 (77.9)	2094 (78.7)	7250 (77.6)	.232
Female	2655 (22.1)	566 (21.3)	2089 (22.4)	
**Race**				
White	7727 (64.4)	1921 (72.2)	5806 (62.2)	< 0.001
Black	1618 (13.5)	217 (8.2)	1401 (15.0)	
Other	2624 (21.9)	513 (19.3)	2111 (22.6)	
Unknown	30 (0.3)	9 (0.3)	21 (0.2)	
**Pathological grade^a^**				
I–II	3545 (29.5)	1109 (41.7)	2436 (26.1)	< 0.001
III–IV	1212 (10.1)	207 (7.8)	1005 (10.8)	
Unknown	7242 (60.4)	1344 (50.5)	5898 (63.2)	
**Surgery**				
Surgery	3736 (31.1)	1147 (43.1)	2589 (27.7)	< 0.001
No surgery	8115 (67.6)	1474 (55.4)	6641 (71.1)	
Unknown	148 (1.2)	39 (1.5)	109 (1.2)	
**AJCC stage^b^**				
I	4087 (34.1)	1224 (46.0)	2863 (30.7)	< 0.001
II	2466 (20.6)	572 (21.5)	1894 (20.3)	
IIIA	2071 (17.3)	333 (12.5)	1738 (18.6)	
IIIB	258 (2.2)	42 (1.6)	216 (2.3)	
IIIC	970 (8.1)	169 (6.4)	801 (8.6)	
IV	2147 (17.9)	320 (12.0)	1827 (19.6)	
**T stage^b^**				
T1	4729 (39.4)	1352 (50.8)	3377 (36.2)	< 0.001
T2	2873 (23.9)	646 (24.3)	2227 (23.8)	
T3	3147 (26.2)	479 (18.0)	2668 (28.6)	
T4	627 (5.2)	88 (3.3)	539 (5.8)	
Tx	623 (5.2)	95 (3.6)	528 (5.7)	
**N stage^b^**				
N0	10014 (83.5)	2346 (88.2)	7668 (82.1)	< 0.001
N1	1405 (11.7)	237 (8.9)	1168 (12.5)	
Nx	580 (4.8)	77 (2.9)	503 (5.4)	
**M stage^b^**				
M0	9367 (78.1)	2262 (85.0)	7105 (76.1)	< 0.001
M1	2582 (21.5)	388 (14.6)	2194 (23.5)	
Mx	50 (0.4)	10 (0.4)	40 (0.4)	

Abbreviations: AJCC, American Joint Committee on Cancer.

a Grade I: well differentiated, II: moderately differentiated, III: poorly differentiated, IV: undifferentiated.

b AJCC Cancer Staging Handbook 6th Edition.

Of the entire study cohort, 2660 patients were AFP negative (A0) and 9339 patients were AFP positive (A1). There were statistically significant differences in all the variables across A0 and A1 stage groups (*P* < 0.05), except sex (*p* = 0.232). Compared with the A0 group, the A1 group had less early cases (fewer grade I and II: 51% vs 67.5%). In addition, more patients in A0 group underwent surgery treatment (43.1% vs 27.7% of A1 group).

### AFP as an independent prognostic factor in HCC

**Figure 1:**
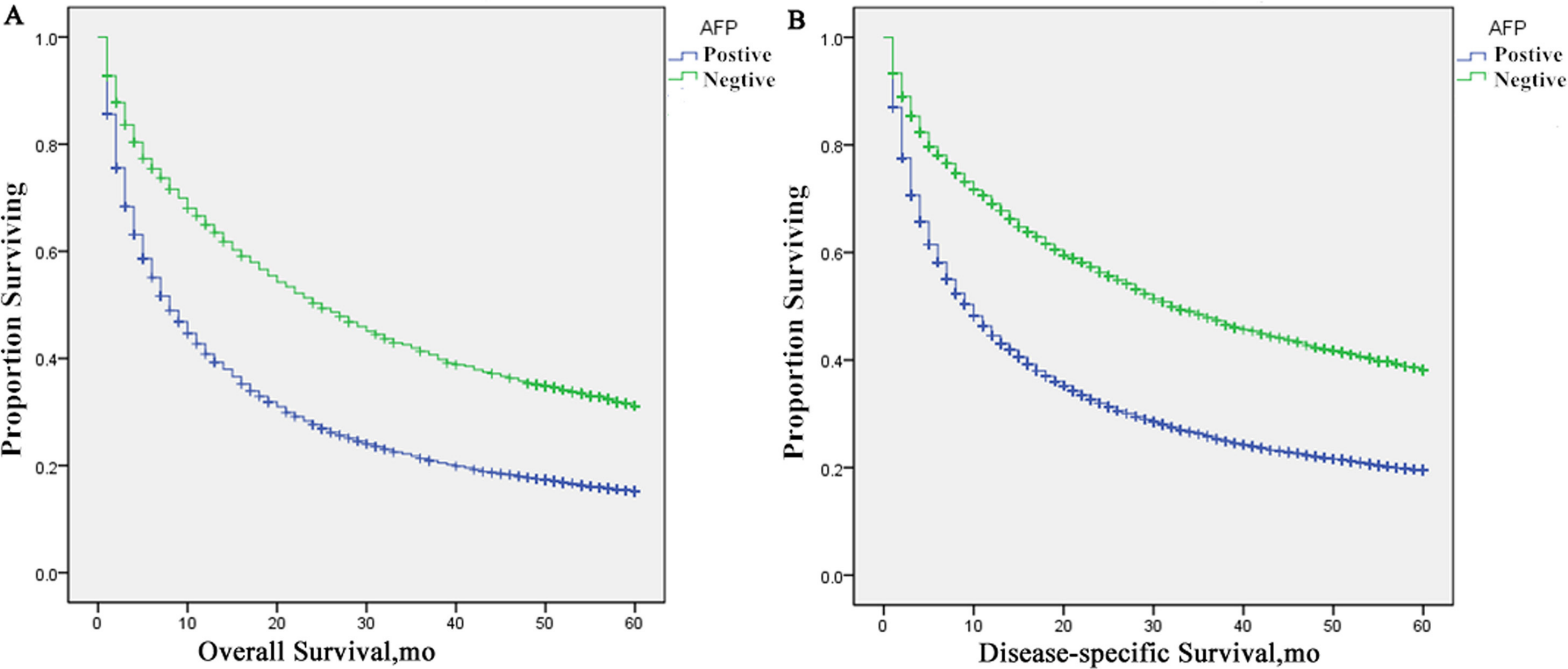
Survival curves in patients according to AFP status via standard Kaplan–Meier estimates (**A**) 5-year OS: AFP positive patients vs. negative patients, χ^2^ = 472.190, *P* < 0.001; (**B**) 5-year DSS: AFP positive patients vs. negative patients, χ^2^ = 477.292 *P* < 0.001.

Multivariate analysis were performed by the Cox regression model to identify factors independently associated with overall and disease-specific mortality (Table 3). AFP positive was an independent poor prognostic factor associated with an increase in overall mortality (HR, 1.460; 95% CI, 1.385–1.539; *P* < 0.001) and disease-specific mortality (HR, 1.514; 95% CI, 1.430–1.603; *P* < 0.001). Moreover, the following four factors were also regarded to be significant risk factors for poorer prognosis, including race (Black, HR 1.115, 95% CI 1.048–1.187; Other, HR 0.822, 95% CI 0.778–0.869), pathological grade (III-IV, HR 1.347, 95% CI 1.245–1.457), T stage (T3, HR 1.748, 95% CI 1.653–1.848; T4, HR 1.676, 95% CI 1.522–1.845; Tx, HR 1.780, 95%CI 0.609–1.968), M stage (M1, HR 1.986, 95% CI 1.862–2.119; Mx, HR 2.277, 95% CI 1.672–3.102). However, no statistical differences were observed with regards to sex (*p* = 0.109), *N* stage (*p* = 0.472) and T2 stage (*p* = 0.559) according to multivariate mortality analysis. The univariate log-rank test showed that the 5-year DSS was 37.65% and 19.23% in the A0 and A1 groups, respectively (*p* < 0.001) (Figure 1).

**Table 3:** Multivariate Cox model analyses for overall and disease-specific mortality

Variable	Overall Mortality		Disease-Specific Mortality	
	HR (95% CI)	*P* value	HR (95% CI)	*P* value
**Year of diagnosis**				
2008	1 [Reference]		1 [Reference]	
2007	1.024 (1.009–1.013)	.424	1.200 (1.120–1.286)	< 0.001
2006	1.068 (1.004–1.135)	.036	1.079 (1.009–1.154)	.026
2005	1.098 (1.032–1.169)	.003	1.059 (0.992–1.130)	.084
2004	1.216 (1.139–1.297)	< 0.001	1.200 (1.120–1.286)	.749
**Sex**				
Female	1 [Reference]		1 [Reference]	
Male	1.068 (1.016–1.122)	< 0.001	1.044 (0.990–1.100)	.109
**Race**				
White	1 [Reference]		1 [Reference]	
Black	1.123 (1.059–1.190)	< 0.001	1.115 (1.048–1.187)	.001
Other	0.823 (0.781–0.866)	< 0.001	0.822 (0.778–0.869)	< 0.001
Unknown	0.728 (0.447–1.108)	.139	0.660 (0.415–1.050)	.079
**Pathological grade**				
I–II	1 [Reference]		1 [Reference]	
III–IV	1.326 (1.230–1.428)	< 0.001	1.347 (1.245–1.457)	< 0.001
Unknown	1.201 (1.144–1.261)	< 0.001	1.199 (1.138–1.263)	< 0.001
**Surgery**				
No	1 [Reference]		1 [Reference]	
Yes	0.770 (0.642–0.924)	< 0.001	0.711 (0.580–0.873)	.001
Unknown	0.362 (0.343–0.383)	.005	0.356 (0.336–0.378)	< 0.001
**AFP stage**				
A0	1 [Reference]		1 [Reference]	
A1	1.460 (1.385–1.539)	< 0.001	1.514 (1.430–1.603)	< 0.001
**T stage**				
T1	1 [Reference]		1 [Reference]	
T2	1.025 (0.970–1.083)	.376	0.982 (0.925–1.043)	.559
T3	1.681 (1.555–1.748)	< 0.001	1.748 (1.653–1.848)	< 0.001
T4	1.593 (1.451–1.748)	< 0.001	1.676 (1.522–1.845)	< 0.001
Tx	1.719 (1.557–1.897)	< 0.001	1.780 (0.609–1.968)	< 0.001
**N stage**				
N0	1 [Reference]		1 [Reference]	
N1	1.052 (0.982–1.127)	.146	1.027 (0.956–1.103)	.472
Nx	1.208 (1.092–1.337)	< 0.001	1.219 (1.101–1.350)	< 0.001
**M stage**				
M0	1 [Reference]		1 [Reference]	
M1	1.757 (1.650–1.870)	< 0.001	1.986 (1.862–2.119)	< 0.001
Mx	2.189 (1.638–2.925)	< 0.001	2.277 (1.672–3.102)	< 0.001

Abbreviations: HR, hazard ratio.

Besides, survival analysis layering for surgical treatment of patients with non-surgical treatment of T3 and T4 patients with different AFP status was conducted via standard Kaplan-Meier estimates, as is shown in Figure S1. In the surgery group, the 5-year OS of T4 with AFP positive was 16.97 months, which is significantly lower than those of T3 stage, 30.5 (*p* < 0.001) in AFP negative and 17.66 (*p* = 0.181) in AFP positive, respectively. However, to the patients with AFP negative was 24 months, which is superior than those of T3 stage, 30.5 (*p* = 0.255) in AFP negative and 17.66 (*p* = 0.285) in AFP positive, respectively (Table S1).

### Prognosis of HCC after incorporation of AFP into TNM staging

Univariate and multivariate analysis were used to analyzed the influences of the incorporation of AFP into the AJCC staging. We calculated the 5-year OS and DSS separately for various AJCC and AFP stage combinations, such as stages I A0, I A1, II A0, II A1, and so on (Table 4). Multivariate analysis was performed by the Cox regression model, as is shown in Table 5. Combined these two tables, we made 2 major observations. First, within each AJCC stage, patients with A1 disease had a worse prognosis compared with those with A0 disease. For example, DSS of stage I A1 was worse than I A0 (31.43% vs 52.8%, *p* < 0.001), and so on. Second, the magnitude of the difference in survival between patients with A0 and A1 disease within each stage was large enough, the prognosis of patients with A1 disease of lower AJCC stages was worse than or similar to that of patients with A0 disease of higher AJCC stages. This finding can be gleaned by observing the overlap of the 95% CIs. For example, disease-specific mortality of patients with stage IIIA A1 (HR, 4.252; 95% CI, 3.839–4.709) is higher than that of patients with stage IIIC A0 (HR, 3.220; 95% CI, 2.654–3.907), and patients with stage IIIC A1 disease (HR, 5.719; 95% CI, 5.092–6.422) is similar to that of patients with stage IV A0 disease (HR, 5.902; 95% CI, 5.121–6.802). I A0, T1 A0 and N0 A0 stages served as control. A similar pattern can be observed in OS outcomes as well.

**Table 4:** Univariate analysis of AFP on OS and DSS based on different stages via standard Kaplan–Meier estimates

Variable	*n*	5-year OS (%)	*P* value	5-year DSS (%)	*P* value
**I**					
A0	1224	41.89	< 0.001	52.80	< 0.001
A1	2863	25.79		31.43	
**II**					
A0	572	38.18	< 0.001	48.64	< 0.001
A1	1894	25.57		34.25	
**IIIA**					
A0	333	13.65	< 0.001	13.65	< 0.001
A1	1738	5.39		7.79	
**IIIB**					
A0	42	17.69	.008	18.16	.028
A1	216	7.80		10.42	
**IIIC**					
A0	169	10.53	< 0.001	13.65	< 0.001
A1	801	4.09		5.90	
**IV**					
A0	320	1.92	< 0.001	1.92	< 0.001
A1	1827	1.61		1.61	
**T1**					
A0	1352	39.24	< 0.001	49.09	< 0.001
A1	3377	22.43		27.26	
**T2**					
A0	646	34.65	< 0.001	43.94	< 0.001
A1	2227	22.44		29.84	
**T3**					
A0	479	9.91	< 0.001	10.32	< 0.001
A1	2668	4.10		5.78	
**T4**					
A0	88	8.28	< 0.001	8.55	< 0.001
A1	539	4.65		5.70	
**N0**					
A0	2346	33.85	< 0.001	41.66	< 0.001
A1	7668	17.66		22.63	
**N1**					
A0	237	9.17	< 0.001	11.19	< 0.001
A1	1168	3.59		4.76	

Abbreviations: OS, overall survival. DSS, disease-specific survival.

**Table 5:** Multivariate Cox model analysis of prognostic factors of HCC on different stages

Variable	*n*	Overall Mortality		Disease-specific Mortality	
		HR (95% CI)	*P* value	HR (95% CI)	*P* value
**I**					
A0	1224	1 [Reference]		1 [Reference]	
A1	2863	1.652 (1.516–1.801)	< 0.001	1.924 (1.744–2.124)	< 0.001
**II**					
A0	572	1.100 (0.966–1.252)	.149	1.123 (0.966–1.304)	.131
A1	1894	1.611 (1.471–1.765)	< 0.001	1.742 (1.568–1.935)	< 0.001
**IIIA**					
A0	333	2.270 (1.968–2.617)	< 0.001	3.080 (2.652–3.576)	< 0.001
A1	1738	3.573 (3.266–3.909)	< 0.001	4.252 (3.839–4.709)	< 0.001
**IIIB**					
A0	42	2.157 (1.529–3.044)	< 0.001	2.839 (1.996–4.037)	< 0.001
A1	216	3.549 (3.025–4.163)	< 0.001	4.415 (3.720–5.239)	< 0.001
**IIIC**					
A0	169	2.758 (2.310–3.293)	< 0.001	3.220 (2.654–3.907)	< 0.001
A1	801	4.767 (4.295–5.291)	< 0.001	5.719 (5.092–6.422)	< 0.001
**IV**					
A0	320	4.395 (3.842–5.028)	< 0.001	5.902 (5.121–6.802)	< 0.001
A1	1827	6.490 (5.929–7.104)	< 0.001	8.550 (7.727–9.460)	< 0.001
**T1**					
A0	1352	1 [Reference]		1 [Reference]	
A1	3377	1.718 (1.587–1.860)	< 0.001	1.962 (1.795–2.146)	< 0.001
**T2**					
A0	646	1.119 (0.994–1.260)	.064	1.142 (0.998–1.307)	.054
A1	2227	1.665 (1.531–1.811)	< 0.001	1.788 (1.626–1.966)	< 0.001
**T3**					
A0	479	2.508 (2.225–2.827)	< 0.001	3.176 (2.798–3.606)	< 0.001
A1	2668	3.705 (3.418–4.017)	< 0.001	4.332 (3.958–4.742)	< 0.001
**T4**					
A0	88	2.585 (2.054–3.254)	< 0.001	3.253 (2.570–4.118)	< 0.001
A1	539	4.331 (3.871–4.846)	< 0.001	5.306 (4.705–5.983)	< 0.001
**N0**					
A0	2346	1 [Reference]		1 [Reference]	
A1	7668	1.711 (1.617–1.810)	< 0.001	1.809 (1.700–1.924)	< 0.001
**N1**					
A0	237	2.252 (1.950–2.600)	< 0.001	2.444 (2.100–2.845)	< 0.001
A1	1168	3.747 (3.463–4.054)	< 0.001	4.122 (3.790–4.483)	< 0.001

Abbreviations: HR, hazard ratio.

Subsequently, we analyzed the association of various combinations of T/N and AFP stages with prognosis to understand the interaction of primary tumor and lymph nodes with AFP status (Table 5). We calculated the hazards for overall and disease-specific mortality along with the respective 95% CIs, compared the outcomes among various combinations. T1 A0 and N0 A0 stages served as control. Again, we observed that patients with A1 disease of each T/N stage had a worse prognosis than patients with A0 disease of the respective stage, and the prognosis of patients with A1 disease with lower T stages was worse or similar to that of patients with A0 disease of higher T stages.

**Figure 2:**
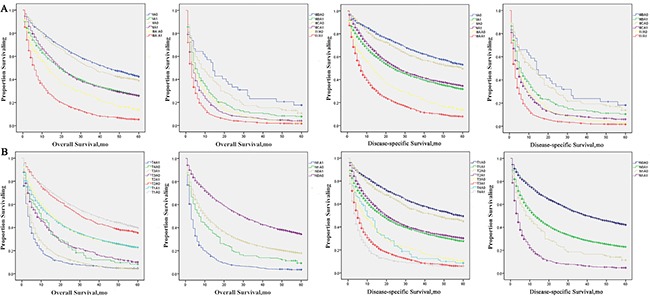
Survival curves in patients according to AFP status combined with each AJCC stage or T/N stage via standard Kaplan–Meier estimates (**A**) 5-year OS and LSS of patients with different combinations of AFP status and each AJCC stage. (**B**) 5-year OS and LSS of patients with different combinations of AFP status and each T/N stage.

This phenomenon is better illustrated in the Kaplan-Meier curves (Figure 2A and 2B), where the curves of patients with A0 disease of certain AJCC stages appear to be grouped or clustered, whereas the curves of patients with C1 disease of the respective AJCC stages appear to be clustered separately.

## DISCUSSION

This study analyzed the long-term prognosis of patients with histologically proven HCC after the incorporation of their pretreatment serum AFP level into the TNM staging system. The large US population-based SEER database was used to collect patient data.

Our previous cohort consisted of 12908 unique records. Nevertheless, the patients with ICC or combined were eliminated after an univariate analysis, because no significant differences (*P* > 0.05) on OS and DSS were observed between A0 and A1 groups of these two histological types. As described by other investigators, HCC tumor markers are AFP and protein induced by vitamin K absence or antagonist II,while ICC tumor markers are carcinoembryonic antigen (CEA) and carbohydrate antigen 19-9 (CA19-9). ICC accounts for approximately 5% of primary liver cancers and AFP positive ICC accounts for less than 1% [13, 14]. Hence, patients with histologically proven HCC were used for statistical analyses.

A prerequisite to proceed with the inclusion of AFP into the staging system is confirmation of AFP as an independent prognostic factor for HCC patients. In this study, we demonstrated that AFP satisfy this prerequisite via multivariate regression analyses. We identified a subset of patients in each AJCC stage by A1 status and noted that OS and DSS of this subset were worse than or similar to a subset of A0 status patients who belonged to a higher AJCC stage. Furthermore, we have proved this phenomenon in the stratified analysis of T stage. These findings indicate the important predictive value of including AFP into the conventional TNM staging and raise the probability of undertreatment of specific HCC patients. Additionally, a global, multicenter, randomized, double-blind, phase 3 REACH study proved that AFP could be a predictive marker for ramucirumab (a second-line treatment in patients with advanced HCC) survival benefit [15]. Similarly, higher AFP level should be considered as an indicator of poor prognosis after liver transplantation [16–18]. Absolutely, AFP seems to be a potential subclassification factor.

However, the patient data extracted from the SEER database was limited to AJCC 6th stage. In 2010, the AJCC updated the arrangement of these TNM combinations [19]. The main variation is the refinement of T3 stage, with redefining T3a as large multinodular and T3b as a major branch of the portal vein or hepatic vein involved. New staging system was regarded to be more active in clinical practice. However, the BCLC is the only staging system with a treatment recommendation based on the stage in West [8]. When integrated into clinical protocols, the results of our study may provide a novel paradigm of HCC management, which depends on the BCLC stage and AJCC stage.

**Figure 3:**
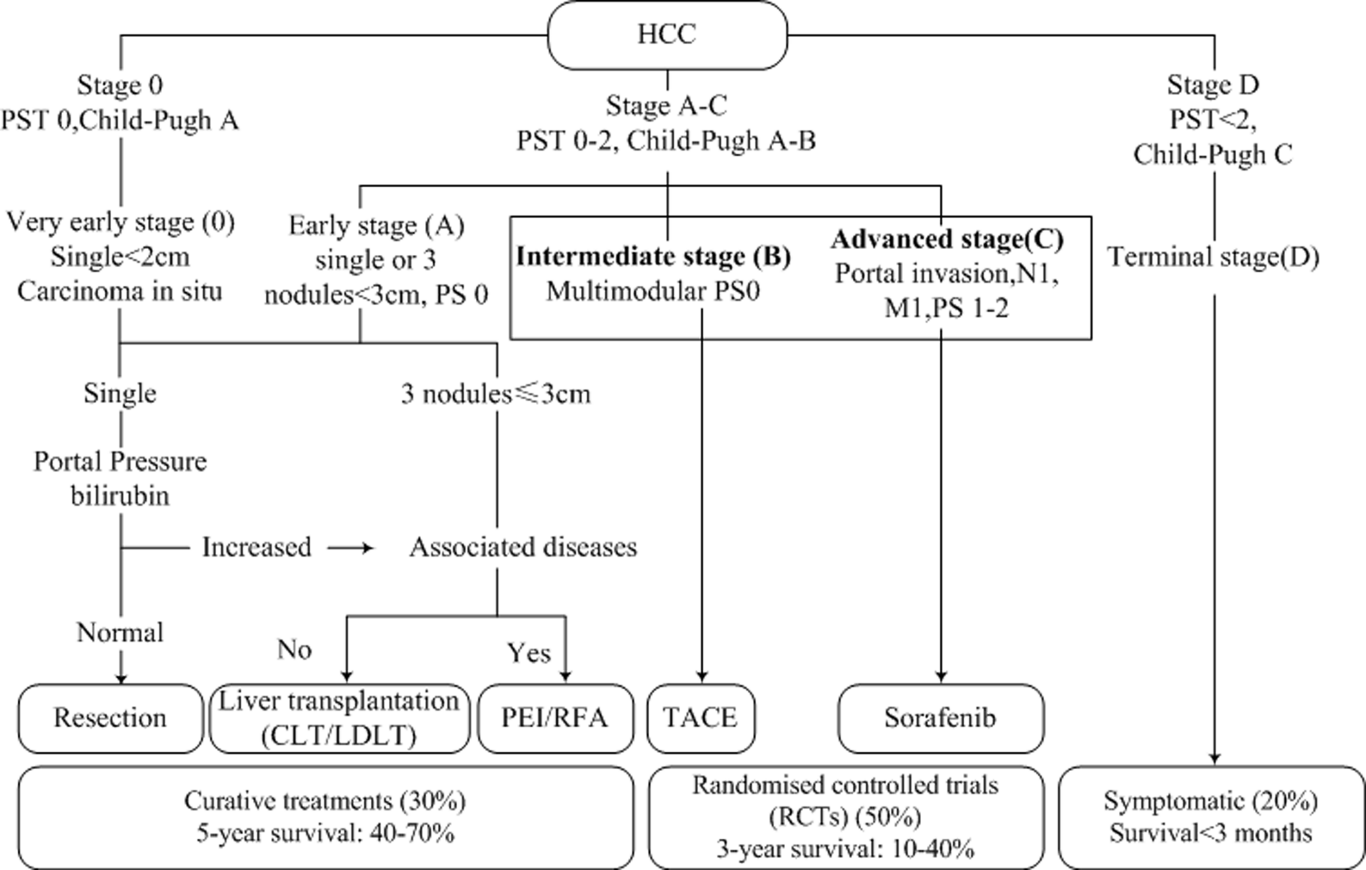
The BCLC stage system and recommended treatment strategies

For further discussion, we analyzed the similarities and differences among the BCLC, the AJCC (6th) and AJCC (7th) staging system. In the United States, the paradigm for treatment of HCC has been BCLC stage specific. Stage 0 is treated with surgical resection alone, stage A with radical therapies (resection, liver transplantation (LT), percutaneous ethanol injection (PEI) or radiofrequency thermal ablation (RFA)), stage B with transarterial chemoembolization (TACE), stage C with new agents in the setting of RCTs (sorafenib, etc.) and stage D with symptomatic treatment (Figure 3) [20]. Following this recommendation, the 5-year OS of the population in stage 0, A was 40–70% without controversy. Nevertheless, new data proved that not following the recommended treatment resulted in a better outcome among the more severe stages (B–D) [8, 20, 21]. Some studies indicated that a more aggressive treatment would yield a better outcome to those patients in stage B or C [22, 23]. In 2012, Bolondi et al. utilized a novel calssification system by modifying the BCLC, especially stage B [24]. Thus, there is controversy regarding the management paradigm of stage B and C, which are discussed in detail below.

According to the AJCC (6th) staging manual, BCLC B is equal to parts of stage T3 and C is equal to part of stage T3 and T4, respectively [25]. Currently, patients with BCLC B are generally treated with TACE, and patients with BCLC C are treated with sorafenib alone. Thus, the decision to recommend radical therapy is often driven mainly by tumor invasion status. In our study, within each T substage, A1 disease predicted poor prognosis compared with patients with A0 disease and the corresponding T stages. Moreover, analysis of every combination of T stage and AFP stage showed that high AFP level disease predicted poor prognosis to a similar magnitude as early T disease. We can conclude that patients with circumscribed HCC with elevated AFP levels may have a poor prognosis similar to those with extra-hepatic spread disease. Therefore, AFP may be integrated into BCLC C stage as a subclassification factor.

Based on these insights and our results, we proposed a subclassification of BCLC B and C stage, as is shown in Table 6. In patients with stage B, we still follow the staging paradigm and recommended treatment proposed by Bolondi et al. However, for patients with stage C, we used a novel subclassification. Patients with A0 in stage C, defined as C1, are suggested to receive resection, leaving patients with moderate to severe cirrhosis. Liu et al. study demonstrated a better overall survival of surgical resection than transarterial chemoembolization (TACE) in HCC patients with BCLC stage C [26]. LT is the optimal treatment even for small, otherwise resectable HCC. However, limited organ availability mandates the restriction of transplantation to only those patients with early stage tumors [27]. Moreover, preoperative treatment can apply to decrease tumor burden to fulfill listing criteria of resection or transplantation and to improve survival. The preoperative treatment includes percutaneous injection of ethanol (PEI) or acetic acid (PAI), RFA, TACE or transarterial embolization (TAE) , radioactive microspheres, and stereotactic radiation. Data suggested that preoperative therapy downstaged the primary tumors and improved survival [28]. To investigate the benefit of surgery treatment for specific population, we conducted the survival analysis layering for surgical treatment of patients with non-surgical treatment of T3 and T4 patients with different AFP status. Our results shows that the 5-year OS of patients in T4S+A+ (T4 patients with surgery treatment and AFP positive) was significantly lower than those of T3S+A+ (T3 patients with surgery treatment and AFP positive) and T3S+A− (T3 patients with surgery treatment and AFP negative). Interestingly, patients in T4S+A− (T4 patients with surgery treatment and AFP negative) group showed similar prognosis on 5-year OS with those in T3S+A+ and T3S+A− groups. Hence, we recommended actively surgery treatment for patients in BCLC C0 stage. Meanwhile, the response to locoregional treatment predicted post-transplant outcome and that this response may be used for patient selection. Furthermore, an AFP level greater than 200 ng/mL was associated with a 3.32-folds increase in the probability of HCC recurrence after LT as reported by Schraiber et al [29]. Besides, transarterial radioembolization (TARE) with yttrium-90 was proved superior to TACE in the treatment of advanced HCC in various studies [30–32]. Percutaneous ablation treatment is not suitable for large HCCs due to the risks of diffusion, vascular washout, and heterogeneity. Patents with A1 in stage C, defined as C2, are suggested to receive new agents in the setting of RCTs (sorafenib, etc.). Sorafenib, an inhibitor of Raf-1, B-Raf, the receptor tyrosine kinases vascular endothelial growth factor receptors (VEGFRs) 1, 2 and 3, and platelet derived growth factor receptor β (PDGFR-β), is now considered as a first-line agent for patients with more advanced HCC. Short-term sorafenib treatment was a potential strategy for recurrence prevention after partial hepatectomy in early-stage HCC. New data has proven its efficacy and safety [33–35]. Moreover, sorafenib is also recommended for patients who have failed TACE or can no longer be treated with more effective therapies [36]. Oherwise, CWP232228, a small molecule inhibitor targeting liver cancer stem cells through Wnt/betacatenin signaling, can also served as a selection [37]. The efficacy and safety of immunosuppression and adjuvant chemotherapy remains to be discussed. In summary, radical treatment may achieve a better prognosis for patients with A0 in BCLC C than traditional BCLC-based recommendation treatment. Further long-term clinical studies remain to be conducted to evaluate and modify the extensional scheme.

**Table 6:** Subclassification and treatment strategy of intermediate and advanced stage HCC

BCLC stage	B				C	
PS	0				1–2	
Tumor size	Large, multinodular			Vascular invasion/Extrahepatic spread		
Okuda stage	I–II				I–II	
Child-Pugh score	5–7		8–9		5–9	
	B1		B2			
Beyond Milan and within up-to-7/AFP	IN	OUT	IN	OUT	A0	A1
**Substage**	**B1a**	**B1b**	**B2a**	**B2b**	**C1**	**C2**
Treatment option	Resection Ablation cTACE	DEB-TACE HAIC Sorafenib	LT Ablation cTACE	HAIC DEB-TACE	Resection/LT^1^ TARE^2^ Sorafenib	Sorafenib CWP232228^3^

Abbreviations: PS, performance scale. LT, liver transplantation.

1 Resection is recommended for those patients leaving moderate to severe cirrhosis. LT is the optimal treatment if organ availability. Preoperative treatments are recommended.

2 TARE can be used as preoperative treatment or for whom surgery is inappropriate.

3 CWP232228 and other new agents in the setting of RCTs.

Taken together, AFP can act as an independent prognostic factor for HCC. Regression analysis indicates that incorporating AFP level into HCC staging system is superior to original AJCC stage in predicting prognosis and selecting more effective treatment. Perhaps more importantly, we can distinguish the population who are more likely to benefit from active treatment and improve survival rate. Much remains to be improved in the staging and treatment of HCC.

Our study mainly investigated the relationship between the expression of AFP and clinical prognosis of HCC patients. We will continue our further research *in vitro*. Some researchers have studied the effects of AFP on liver cancer cell growth, migration, and apoptosis. Over the past few decades, professor Mengsen Li with his partners have been addressing the connection between AFP and liver cancer cell growth, apoptosis and drug resistance. Their studies demonstrated that AFP and AFPR may play pivotal role in HBV-related malignant transformation of hepatocytes via the activation of PI3K/mTOR signaling pathway. Actuated expression of AFPR plays a role of an indicator suitable for use in the early diagnosis of HBx-driven malignant transformation of hepatocytes. Labeled AFPR is likely to trace primary and metastatic HCC [38, 39]. Furthermore, AFP is an important molecule acting against paclitaxel-induced proliferation inhibition and apoptosis in HCC cells, which indicated that inhibiting AFP expression after treatment with paclitaxel may be an available strategy for the treatment of HCC [40]. Besides, AFP plays a crucial role in promoting metastasis of HCC via up-regulating expression of metastasis-related proteins [41].

The AFP could be a regulator in the phosphatidylinositol 3-kinase (PI3K)/protein kinase B (AKT) pathway, which has been proven to be a key upstream signaling pathway of EMT [42]. In addition, the activation of PI3K/mTOR signaling pathway has been proven to be related with HBx-induced AFP expression and subsequently promotes malignant transformation in liver cells [38]. Hence, regulation of PI3K/AKT signaling pathway may be the possible molecular mechanism that mediate AFP-induced malignant progression of liver cancer, such as tumorigenesis, growth, migration and invasion. We will investigate it in our further research.

A major limitation of our study is that the influence factor of HCC prognosis is variable, such as the Albumin-Bilirubin (ALBI) score [43], tumor differentiation [44], race, pathogenic factors, and so on. Many other subclassifications of BCLC B and C stages have been proposed recent years. If possible, these factors are essential to be included in the staging model. Additionally, the clinical utility of our recommendation is still looking forward to be validated via prospective randomized studies.

In conclusion, the management of HCC is still a great challenge for clinicians. AFP, an independent prognostic factor, can be incorporated into the BCLC staging system to modify the recommended treatment of patients with stage C. Further study is needed to prospectively determine the clinical utility of this modified BCLC staging system.

## MATERIALS AND METHODS

### Data collection and patient selection

This study was based on public data from the SEER database; we obtained permission to access research data files with the reference number 13895-Nov2014. Given that these data are deidentified and ethics approval is waived, the study did not require informed consent, and was approved by the Review Board of Nanjing Medical University (Nanjing, China).

The SEER Cancer Statistics Review (http://seer.cancer.gov/data/citation.html), which is available for studies of cancer-based epidemiology and survival analysis, is published annually by the Data Analysis and Interpretation Branch of the National Cancer Institute (Bethesda, MD, USA). The SEER database includes approximately 26% of the population in the USA. Using SEER*Stat, an online access program provided by the SEER program, We extracted data from January 1, 2004, through December 31, 2008, using the National Cancer Institute's SEER*Stat software (http://seer.cancer.gov/seerstat) (Version 8.2.1).

All patients were pathological diagnosed as liver cancer based on International Classification of Diseases for Oncology, Third Edition (ICD-O-3), morphological codes (C22.0 and C22.1). Only patients with available pretreatment serum AFP level information were included. Furthermore, we included data pertaining to age; sex (male and non-pregnant female); race; histological type; TNM stage; cause-specific death classification; end calculated vital status; overall survival (OS); disease-specific survival (DSS); and use of surgical resection. All AFP level information were coded as “test not done”, “positive/elevated”, “negative/normal; within normal limits”, “borderline; undetermined whether positive or negative”, “ordered, but results not in chart” or “unknown or no information”. Patients were excluded if they had no conclusive evaluation of pretreatment serum AFP level. We designated stage A0 for patients with a serum AFP level coded as “negative/normal; within normal limits” (AFP 0–15 ng/mL) and A1 for those coded as “positive/elevated” (AFP > 15 ng/mL). All survival estimates were based on data from the SEER database. All patients were staged according to the 6th AJCC Cancer Staging Handbook [39]. Further details about the data were obtained according to the SEER Data Management System User Manual (http://seer.cancer.gov). Death was treated as events. Accordingly, alive or deaths from other causes were treated as censored observation. The primary endpoint of the study is DSS, which was calculated from the date of diagnosis to the date of disease-specific death.

### Statistical analyses

The overall survival (OS) and disease-specific survival (DSS) (ie, liver cancer) curves of all stage groups were generated using standard Kaplan–Meier estimates, respectively. Differences among the curves were analyzed by log-rank test in a pairwise fashion. Univariate and multivariate Cox regression models were built to obtain hazard ratios (HRs) of risk factors for survival outcomes. We used two novel covariates (NCs), which are combination of T/N and AFP stage to study the interaction of these covariates on prognosis. All statistical analyses were conducted using SPSS statistical software, version 19 (SPSS Inc., Chicago, IL, USA). *P* < 0.05 were considered statistically significant.

## SUPPLEMENTARY MATERIALS


