# Supplementary material for: Incorporation of alpha-fetoprotein(AFP) into subclassification of BCLC C stage hepatocellular carcinoma according to a 5-year survival analysis based on the SEER database
Source: Oncotarget. 2016 Nov 9;7(49):81389–401. doi: 10.18632/oncotarget.13232 (PMC5348400; doi:10.18632/oncotarget.13232)
Supplement: Supplementary file 1 [file oncotarget-07-81389-s001.pdf]

Incorporation of alpha-fetoprotein(AFP) into subclassification of BCLC C stage hepatocellular carcinoma according to a 5-year survival analysis based on the SEER database

Supplementary Materials

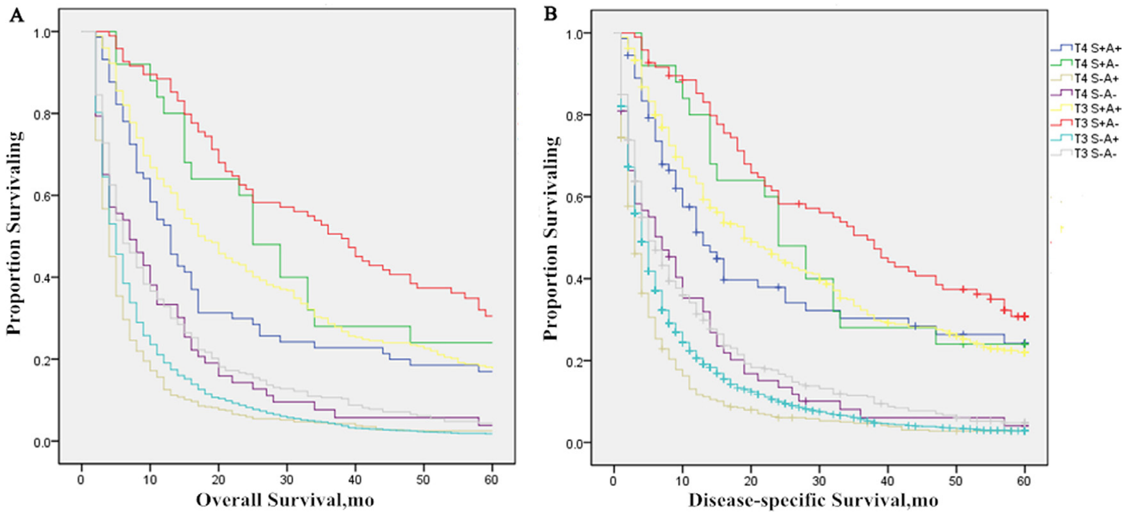

Supplementary Figure S1: Survival analysis layering for surgical treatment of patients with non-surgical treatment of T3 and T4 patients with different AFP status was conducted via standard Kaplan-Meier estimates. T3 and T4 patients were divided into eight groups: T3 patients with surgery treatment and AFP negative as T3S+A-; T3 patients with non-surgery treatment and AFP positive as T3S-A+; T3 patients with surgery treatment and AFP positive as T3S+A+; T3 patients with non-surgery treatment and AFP negative as T3S-A−; T4 patients with surgery treatment and AFP negative as T4S+A−; T4 patients with non-surgery treatment and AFP positive as T4S-A+; T4 patients with surgery treatment and AFP positive as T4S+A+; T4 patients with non-surgery treatment and AFP negative as T4S-A-. (**A**) 5-year OS; (**B**) 5-year DSS.

Supplementary Table S1: The 5-year OS and DSS of T3 and T4 stage patients layering for surgical treatment of patients with non-surgical treatment

Variable	*n*	5-year OS (%)	*P* value	5-year DSS (%)	*P* value
**T3(Surgery)**					
A0	96	30.50	< 0.001	30.50	.003
A1	373	17.66		21.83	
**T3(no surgery)**					
A0	379	4.39	< 0.001	4.70	< 0.001
A1	2263	1.78		2.74	
**T4(Surgery)**					
A0	25	24.00	.073	24.00	.316
A1	73	16.97		23.99	
**T4(no surgery)**					
**A0**	63	3.81	.004	2.76	.003
**A1**	461	2.49		4.02	

Abbreviation: OS, overall survival. DSS, disease-specific survival.
